# Ngā māuiui kai: a cross-sectional study of elevated eating disorder risk and related experiences among trans people in Aotearoa

**DOI:** 10.3389/fpsyt.2026.1804270

**Published:** 2026-04-27

**Authors:** Alex Ker, Gloria Fraser, Micah Davison, Jaimie Veale

**Affiliations:** 1 School of Health, Te Herenga Waka – Victoria University of Wellington, Wellington, New Zealand; 2 Alumni of the School of Health Sciences, University of Canterbury, Christchurch, New Zealand; 3 School of Psychology, University of Waikato, Hamilton, New Zealand

**Keywords:** disordered eating, eating disorder, healthcare access, LGBTQIA, mental health, transgender

## Abstract

**Purpose:**

Little is known about disordered eating and eating disorders (ngā māuiui kai) among transgender and non-binary (trans) communities in Aotearoa New Zealand. This cross-sectional study sought to provide evidence of the prevalence and experiences of ngā māuiui kai among these communities.

**Methods:**

We analyzed data from a national trans health survey of people using chi-square tests of independence to examine associations between sociodemographic characteristics and elevated eating disorder risk measured by the SCOFF screening tool. A content analysis of open-text survey comments identified themes across participants’ self-reported experiences of ngā māuiui kai.

**Results:**

Overall, 34.3% of participants met criteria for increased risk for an eating disorder. Age, neurodivergence, material hardship, functional impairment, and Māori ethnicity were associated with elevated risk among this sample. No associations were found for gender, self-identified disability, or other ethnicities. The content analysis found that several participants reported connections between their māuiui kai and gender incongruence, broader mental health issues, or structural barriers. Some reported challenges seeking related healthcare, and a lack of providers’ awareness of the relationship between gender-affirming healthcare needs and ngā māuiui kai.

**Conclusions:**

A high proportion of trans participants met the criteria for elevated risk of eating disorders, with higher risk among those belonging to other marginalized groups. These findings highlight the unique risk factors among trans people who belong to multiple marginalized groups. They signal need for appropriate prevention and provision of responsive care for trans people at the intersections of ngā māuiui kai and gender-affirming healthcare.

## 1 Introduction

Eating disorders and disordered eating encompass a range of difficulties related to food, eating behaviors, and body image, with significant impacts on mental and physical health outcomes (1–3). Eating and body image difficulties are shaped by broader sociocultural forces, including appearance ideals and systemic inequities, and are not experienced equally across population groups (4–7). In this article, we use the term “ngā māuiui kai”, an Indigenous te reo Māori term meaning to be “out of balance or out of sorts in relation to food” (8, p. 4) to encompass both formal diagnosis and so-called “subclinical” presentations of eating disorders and disordered eating.

Prevalence data of ngā māuiui kai among general populations in Aotearoa is limited. Te Rau Hinengaro, the New Zealand Mental Health Survey, reported lifetime prevalence rates of 1.7% for eating disorders using DSM-IV criteria (0.6% for anorexia nervosa and 1.3% for bulimia nervosa; 9). More recent data from the World Health Organization’s World Mental Health Surveys reported a lifetime prevalence of 1.3% for bulimia nervosa and 1.9% for binge eating disorder in Aotearoa (10). Te Rau Hinengaro excluded participants under 16, and the WHO survey excluded those under 18, despite eating disorders frequently emerging in childhood and adolescence (11).

More recent evidence from specialist eating disorder service referrals and hospitalizations suggests that the prevalence of ngā māuiui kai has risen in Aotearoa since the COVID-19 pandemic (12). These administrative data sources did not use trans-inclusive data collection measures, limiting their relevance for transgender and non-binary (trans) communities in Aotearoa. Recent research found that one third of trans youth in Aotearoa met the threshold for being at risk of an eating disorder (13). However, no published research in Aotearoa to date has examined trans people’s experiences across the full life span.

### 1.2 Ngā māuiui kai among trans people

International data shows that trans people report ngā māuiui kai symptoms at rates two to four times higher than cisgender participants (14–21). Estimates indicate that 20-50% of trans people develop disordered eating behaviors in their lifetime, and 2-12% of trans people report having been diagnosed with an eating disorder (20, 22). The factors contributing to ngā māuiui kai among trans people are likely to reflect the same broad biopsychosocial factors seen in the general population, alongside additional considerations that may place some trans people at elevated risk.

One contributing factor to ngā māuiui kai among trans people is the complex relationship between eating behaviors, body image, and gender dysphoria – the distress that can arise from the incongruence between one’s gender and sex assigned at birth (20, 23, 24). This distress often occurs within societal contexts that marginalize and pathologize trans people’s bodies (25). Some trans people report engaging in disordered eating to manage gender-related distress, regulate negative affect associated with their bodies, suppress secondary sex characteristics, or achieve a body that aligns more with their gender identity (20, 26, 27). However, qualitative studies also show that some trans people understand their eating issues to be separate from their gender-related distress (23, 27).

These varied findings suggest that eating difficulties among trans people cannot be explained by gender dysphoria alone. Minority stress—chronic stress resulting from experiences of discrimination, invalidation, and social exclusion—is well established as a key social determinant of mental health difficulties among trans people (28, 29). Intersecting factors such as racism, lack of support, and economic and food insecurity may further shape how trans people experience, interpret, and respond to ngā māuiui kai (30–34). Further, clinicians often lack the knowledge and skills required to meet trans people’s needs (35). Taken together with these clinical and structural barriers, assumptions about who experiences ngā māuiui kai may mean trans people are not identified as experiencing these symptoms, or they may be less likely to seek care (30). Overall, existing research suggests that reasons for developing ngā māuiui among trans people are varied, multifactorial, and context-dependent.

Understanding these diverse experiences is important for developing responsive and inclusive interventions to help trans people seek support, manage their symptoms, and recover from ngā māuiui kai. The research gap in Aotearoa in this area is particularly concerning given the growing awareness of mental health inequities experienced by trans communities. This includes high rates of psychological distress, suicidality, and discrimination in healthcare settings (36, 37), alongside a lack of tailored ngā māuiui kai interventions. This is the first study in Aotearoa to bring together prevalence estimates and qualitative accounts of ngā māuiui kai among trans people across the lifespan.

## 2 Materials and methods

In this descriptive cross-sectional study, we sought to (1) estimate the elevated risk of eating disorders within trans communities in Aotearoa, (2) explore the sociodemographic characteristics associated with elevated risk, and (3) better understand how trans people make sense of their experiences of ngā māuiui kai in relation to their gender and broader mental health.

To address these aims, we analyzed data from the second iteration of Counting Ourselves, a national community-based, non-randomized survey about trans people’s health in Aotearoa (37, 38). The online survey, asking a range of closed- and open-text questions about health and wellbeing in English, was open between 1 September and 14 December 2022.

Participants were invited to take part through various purposive strategies including outreach from community leaders and organization’s, efforts to reach Māori, Pacific, Asian, older and rural trans people. Recruitment materials were translated into multiple languages and shared through email listservs and social media posts. People were eligible to take part if they were trans or non-binary (i.e., their gender differed from their sex assigned at birth), 14 years or older, and lived in Aotearoa at the time of taking the survey. Participants gave consent by beginning the survey after reading participant information. After data cleaning, the final sample for analysis was *N* = 2631 (Mean age = 27, SD = 11.7). Counting Ourselves received ethics approval from the Health and Disability Ethics Committee, reference 2022 FULL 12683.

The co-authors of this study are a group of both trans and cis, and both Māori and Pākehā/NZ European researchers. Our range of related personal, professional and clinical experiences of living with and working with ngā māuiui kai have informed our approach to the study design and analysis. Throughout the analysis, we engaged in reflective discussions on how our positions, and related assumptions about ngā māuiui kai, may have influenced our coding and interpretation of findings. Our knowledge in this area helped to interpret data in a more nuanced way, while combining our diverse experiences helped to cross-check for any biases or “analytic blind spots” (39) that may have influenced our interpretation of the data. Authors involved in the analysis kept memos throughout analysis to discuss with the wider team.

### 2.1 Measures

#### 2.1.1 Elevated risk of eating disorder (dependent variable)

The Sick, Control, One Stone, Fat, Food (SCOFF), a five-item validated screening tool (22, 40), was used for its brevity to determine elevated risk of an eating disorder. Participants could respond ‘Yes’ or ‘No’ to each of the items. In line with Coop et al.’s (41) guidance, participants’ scores were added and binarized so that participants who answered ‘Yes’ to two or more items were coded as meeting the threshold for an elevated risk of eating disorder (1), and participants who said yes to one item or none, were coded as not meeting the threshold (0).

#### 2.1.2 Sociodemographic characteristics (independent variables)

##### 2.1.2.1 Gender

Gender was measured as a categorical variable using the item, ‘Recognizing that we are giving you limited options, if you had to select one response that best describes your current gender (or equivalent gender in English), what would it be?’ Response options included ‘Trans woman or girl’; ‘Trans man or boy’, or ‘Non-binary, genderqueer, agender, or similar identity’. These three categories were used as gender groups for analysis.

##### 2.1.2.2 Age group

Participants were asked ‘What is your age in years?’ Three age groups were created for analysis: youth (14–24 years), adult (25–54 years), and older adult (55+ years).

##### 2.1.2.3 Ethnicity

We used five categories derived from Stats NZ’s (2005) Level 1 Ethnicity New Zealand Standard Classification: Māori; Pacific; Asian; European; and Middle Eastern, Latin American, African (MELAA) or another ethnicity. Each ethnic group was binarized and tested individually, such that those who had selected the respective ethnic group were in the reference group (i.e., Māori = 1; non-Māori = 0).

##### 2.1.2.4 Functional impairment and disability

Participants were identified as disabled if they self-identified as Deaf or disabled, and/or if they met the criteria for having a functional impairment based on the Washington Group Short Set (WG-SS) items (Washington Group on Disability Statistics, n.d.). In this article we report self-identified disability and functional impairment separately.

##### 2.1.2.5 Autism or ADHD

Participants self-identified if they had autism or ADHD (with or without formal diagnosis) in the survey. We did not rely exclusively on formal diagnosis due to the structural barriers that can prevent people from seeking assessment and diagnoses for autism and ADHD.

##### 2.1.2.6 Material hardship

Seven items adapted from the DEP-17 index (42) were asked in Counting Ourselves about things participants did to keep costs down over the previous 12 months (e.g., “gone without fresh fruit or vegetables”). Proportional to the cut-off used in the original index (≥6 out of 17 items), participants who selected three or more of these items were classified as experiencing higher material hardship, while those who selected two or fewer items were classified as experiencing lower material hardship.

### 2.2 Analysis

Data were cleaned prior to analysis by members of the wider Counting Ourselves team. Descriptive statistics analyses were conducted on IBM SPSS Statistics v30 for Windows. Little’s MCAR (missing completely at random) test of SCOFF non-responses indicated missing data was likely random (*p* = .253). Missing SCOFF data were therefore excluded from the analysis. A series of chi-square tests of independence were first conducted to identify strength of associations between each sociodemographic variable (gender, age, ethnicity, material hardship, disability, and neurodivergence) and threshold for elevated risk of an eating disorder (SCOFF). Cramer’s V was used to measure effect sizes for variables with more than two categories (age and gender), and Phi was used for binary variables (disability, functional impairment, ADHD, autism, ethnicity, and material hardship). Following Cohen’s (43) guidance for chi square analyses, effect sizes are considered small at.1, medium at.3, and large at.5.

To gain further insight into participants’ experiences of ngā māuiui kai, we analyzed open-text responses from survey questions asking about mental health using conventional descriptive content analysis (44). Our pragmatic and inductive approach to analysis was suited to our research aims of describing high-level patterns across data related to ngā māuiui kai, to identify areas for further in-depth research. The analysis was led by the first author, AK, who met with GF and MD to discuss the coding process. AK first read through all survey open-text responses to identify which responses contained references to ngā māuiui kai including food, eating, weight, disordered eating, and eating disorders.

Overall, 36 individual participants provided 47 responses relating to these topics, across 18 open-response questions inviting participants to share their mental health and healthcare experiences in more detail. AK then collated and familiarized himself these comments. He then coded the data, labelling units of data with both latent (e.g., ‘weight stigma’) and manifest (e.g., ‘Eating is a way of coping with gender dysphoria’) codes. After an initial round of coding and preliminary categorization, AK and MD then met to discuss the and refine coding and categories where necessary. MD then coded a sample of quotes, and we finalized the agreed codes and thematic categories. We were not able to member-check as survey responses were anonymous.

## 3 Results

### 3.1 Chi square test of independence results

Overall, 77.3% (*n* = 2035) of all Counting Ourselves participants answered the SCOFF questionnaire. Just over one-third (34.3%; *n* = 697) met the threshold for elevated risk of an eating disorder. The chi square tests of independence showed that elevated risk of an eating disorder among these participants was significantly associated with age *X^2^*(2 *n* = 2035) = 31.24, *p* = <.001, having a functional impairment *X^2^*(1 *n* = 1940) = 90.90, *p* = <.001, material hardship *X^2^*(1 *n* = 1716) = 41.14, *p* = <.001, autism *X^2^*(1 *n* = 2003) = 8.84 *p* = .003, ADHD *X^2^*(1 *n* = 2003) = 15.08, *p* = <.001, and Māori ethnicity *X^2^*(1 *n* = 2030) = 12.49, *p* = <.001. All effect sizes for significant associations were small (i.e., <0.30; see **Table 1**).

**Table 1:** Sample characteristics of trans people in Aotearoa by SCOFF threshold, 2022 Counting Ourselves (n=2035).

Characteristic groups	*n* (%) within group who meets the SCOFF threshold (*n* = 697)	Effect size^a^	Chi-square^b^
Age		.124	***p* <**.**001**
Youth (14-24)	398 (39.4%)		
Adult (25-54)	282 (30.5%)		
Older adult (55+)	17 (16.8%)		
Gender		.044	*p=*.147
Trans man	161 (36.2%)		
Trans woman	142 (30.5%)		
Non-binary	385 (35.0%)		
Ethnicity^c^			
Māori	113 (44.1%)	.078	***p* <**.**001**
Pacific	19 (47.5%)	.039	*p=*.077
Asian	45 (31.3%)	-.018	*p=*.426
NZ European	518 (33.1%)	-.043	*p=*.055
MELAA or another ethnicity	14 (31.1%)	-.010	*p=*.654
Functional impairment (WGSS)	346 (46.9%)	.216	***p* <**.**001**
Deaf or disabled (self-identified)	224 (37.7%)	.043	p=.058
ADHD	310 (39.6%)	.087	***p* <**.**001**
Autism	286 (38.9%)	.066	***p=*** **.003**
Material hardship	459 (39.0%)	.138	***p* <**.**001**

^a^Cramer’s V was used to measure the effect size of gender and age (larger than 2x2 contingency tables). Phi was used to measure effect size of all other sociodemographic variables.

^b^Bold *p*-values indicate significant at *p ≤*0.05.

^c^Ethnicity was measured as a total count, rather than being prioritised. Each ethnicity category was tested compared to a binary reference group (ie. Māori, non-Māori).

Conversely, no significant associations were found between elevated risk for eating disorders and gender *X^2^* (2 *n =* 2011) = 3.835 *p = .*147, self-identifying as Deaf or disabled *X^2^*(1 *n* = 1988) = 3.604, *p* = .058, and Asian *X^2^* (1 *n* = 2033) = .633, *p = .*426, Pacific, *X^2^* (1 *n* = 2030) = 3.137, *p = .*077, European *X^2^* (2 *n =* 2035) = 3.6582 *p = .*055, or another ethnicity *X^2^* (1 *n =* 2035) = .201 *p = .*654.

### 3.2 Content analysis findings

We identified five interrelated themes from our content analysis (**Table 2**). The most common factor participants attributed to their māuiui kai was their experience of gender incongruence. Some described how their eating challenges began around puberty, and described how their eating disorder was “enmeshed” with their gender exploration. For example, one participant wrote “*everyone assumed that [my eating disorder] was due to other factors, but the truth is that it was to try and stop puberty happening”.*

**Table 2:** Themes from conventional content analysis of open-text responses on ngā māuiui kai^a^ (responses related to disordered eating, eating disorders, food, body image, and weight).

Theme	Description	Example quotes
Ngā māuiui kai is related to gender incongruence	Many participants (*n* = 15) shared their māuiui kai was connected to their experiences of being trans, gender dysphoria, or the onset of puberty. Some understood their eating disorder as a way of coping with their dysphoria through suppressing secondary sex characteristics.	*My experience working things out with my gender has been quite enmeshed in my eating disorder. I developed anorexia right at puberty, age 14. I think this was quite linked in hindsight to being trans. Didn’t have the awareness or language for it at that time though.* *I have had a history of eating disorders since i started puberty - everyone assumed that it was due to other factors, but the truth is that it was to try and stop puberty happening. I struggled with having a female body for the entirety of my adult life. Having chest reconstruction and a hysterectomy has literally changed my life! It is SUCH a relief to not be afraid of my own body.*
Ngā māuiui kai is related to broader mental health challenges	Participants (*n* = 13) commented that their eating issues were related to other aspects of their mental health, such as depression and trauma.	*I used to self-harm and engage in negative eating patterns with food as a means of control. One of the hardest symptoms of C-PTSD is suicidal ideation. When I didn’t eat, I felt these feelings of harm and self-destruction the most* *Being trans helps me love myself and feel like live is worth living. My issues with food feel like an effect of broader mental health issues rather than a* sp*ecific eating disorder.*
Social and economic factors contributing to ngā māuiui kai	Some participants (*n* = 8) identified external or structural factors that contribute to how they experience ngā māuiui kai and mental health, including gendered assumptions about weight and bodies, marginalization, hostile messages about trans people, and not being able to afford kai.	*It is hard to have hope when the whole medical system is against you because of your weight, but one of the ways you cope with your dysphoria is through eating.* *A lot of it compacts with being poor and an unaccepting environment. When I have more financial freedom (i.e. can afford a roof and food) and have time to socialize with trans accepting people instead of always working, I’m healthier.*
Challenges accessing trans-knowledgeable care for ngā māuiui kai	Some participants (*n* = 9) described challenges in seeking care for ngā māuiui kai as a trans person. Participants who shared care-seeking experiences encountered varying levels of trans awareness in related healthcare services. Some reported not being able to access care when they needed it due to long wait times, or not being able to find providers who understood the relationship between gender diversity and ngā māuiui kai.	*There was really no precedent for someone being non-binary in the services so when I came out to my support worker they were unable to address any of the ways that being non-binary contributed to my ED.* *I hope services for eating disorders in future will be more competent working with trans people. At this point, there is treatment for being trans OR having and [sic] ED. Nothing really that ties the two together.*
Navigating ngā māuiui kai and gender-affirming healthcare	Several participants (*n* = 5) described how ngā māuiui kai were exacerbated by negative healthcare experiences when accessing gender-affirming healthcare, such as being told to lose and keep off weight before getting top surgery. Some shared negative experiences of mental health professionals not understanding the complex relationship between the importance of accessing care for both ngā māuiui kai and gender affirmation. Some participants described that affirming their gender helped them connect to their body.	*The waitlist to see the eating disorder* sp*ecialist was extremely long, and the psychiatrist wouldn’t refer me to the top surgery waiting list until I provided her a letter from a different therapist* sp*eaking of my need for the surgery. This delayed the waitlist process by three months, which was extremely tedious, as I was already having significant health problems from binding and using trans tape.*

^a^Ngā māuiui kai is an Indigenous te reo Māori term to encompass both formal diagnosis and so-called “subclinical” presentations of eating disorders and disordered eating.

Other participants shared ngā māuiui kai was related more broadly to mental health challenges, as a way of managing difficult emotions or trauma. Structural drivers, including weight stigma and food insecurity, were also reported by a few participants, although these were less common.

Two further themes relate to challenges accessing healthcare for ngā māuiui kai and gender-affirming healthcare. Participants who shared care-seeking experiences for ngā māuiui kai encountered varying levels of trans awareness in mental health services. Long wait times, or difficulty finding providers who understood the relationship between gender diversity and ngā māuiui kai were common barriers to accessing care. As one participant shared: “*I hope services for eating disorders in future will be more competent working with trans people”.*

In addition, participants described limited awareness among providers of the relationship between accessing gender-affirming healthcare and recovering from ngā māuiui kai. Some described difficulty accessing care for both concurrently: “*the [eating disorder] psychiatrist wouldn’t refer me to the top surgery waiting list until I provided her a letter from a different therapist* sp*eaking of my need for the surgery”.*

While Māori ethnicity was found to be associated with elevated risk of ngā māuiui kai, the relationship between being Māori and experiencing eating-related challenges, or broader experiences at the intersections of ethnicity, culture, or race was not reflected in open-text comments. Together, these themes suggest that trans people’s experiences of ngā māuiui kai are multifaceted and often, but not always, related to their gender and need for gender-affirming healthcare.

## 4 Discussion

The purpose of this study was to describe ngā māuiui kai among trans communities in Aotearoa, and to advance preliminary understandings of who among trans communities is at elevated risk of developing an eating disorder. We contextualized these quantitative findings using qualitative accounts from participants who shared experiences of ngā māuiui kai.

Overall, a high proportion of participants met the threshold for elevated eating disorder risk. These prevalence levels are similar to those reported in previous studies using the SCOFF with trans populations, including research showing that 28% of trans youth (45) and 34%-38.8% of trans college students (19) reported elevated risk. Similar to our findings, no differences of risk were found between gender groups in Linsenmeyer and colleagues’ (2021) study. While the prevalence of risk among trans women in Simone and colleagues’ (2022) study was not significantly different from that of trans men or gender nonconforming or expansive college students, trans men reported significantly lower risk relative to gender nonconforming or expansive students. Further, research using other measures of ngā māuiui kai among trans people has identified gender-related differences in symptoms (e.g., 16, 33). This indicates that the way ngā māuiui kai is measured may influence whether differences across gender groups in trans populations are detected, and that there may be contributing factors unique to people who share similar gender trajectories. These mixed patterns also contrast with general population data, where cisgender women have markedly higher rates of eating disorders than cisgender men (46).

Our findings challenge narrow stereotypes of who experiences ngā māuiui kai. The idea that eating disorders primarily affect “skinny, white, affluent girls” (SWAG; 47) does not align with our results. Instead, significant associations with age, functional impairment, some types of neurodivergence, material hardship, and Māori ethnicity suggest that trans people who belong to these groups experience compounding risk factors. The stronger association between functional impairment, compared with self-identified disability, suggests how disability is defined influences observed patterns, and should be examined further.

From an intersectional perspective (see 48), these findings may reflect the interaction of multiple systems of oppression – including racism, ableism, classism, transphobia, cisnormativity – in shaping unequal health outcomes. For example, Lacey and colleagues (2020) have suggested that higher rates of lifetime prevalence of ngā māuiui kai among Māori may be attributable to systemic bias in social and healthcare services, contributing to disproportionately lower rates of referrals to specialist services. Although almost half of Pacific respondents in our study reported elevated risk, the association did not reach statistical significance, which is likely due to small sample size of Pacific participants overall. The absence of national prevalence data for ngā māuiui kai in the general populations in Aotearoa also limits comparison across groups.

The qualitative themes show that puberty and gender incongruence were important factors in the development of ngā māuiui kai for some participants. These findings support the quantitative association between younger age and elevated risk, highlighting this developmental stage as a critical point for developing interventions that address both care for gender dysphoria and ngā māuiui kai. Previous qualitative research suggests that ngā māuiui kai can be more related to broad mental health challenges that are not directly related to gender (23, 27). Participants also described barriers to accessing care for ngā māuiui kai, including long wait times and variable levels of trans awareness among providers. These experiences indicate an unmet need for specialist services, although rates of trans people accessing specialist services are currently not known due to lack of accurate data collection.

Combined with our findings of elevated risk among trans people who hold dual or multiple marginalized identities, suggests the importance of healthcare professionals to ask trans clients how they understand the factors contributing to their māuiui kai and their needs or goals around gender affirmation, rather than assuming what these might be. While our quantitative results found associations between Māori ethnicity and elevated risk, this was not reflected in the qualitative data. This likely reflects the small number of open-text responses and the absence of dedicated questions about ngā māuiui kai. Further research is needed to understand the experiences of Maori trans people, and the role of culturally grounded approaches to care.

Understanding the factors that contribute to the development of, and recovery from, ngā māuiui kai among trans people may enable healthcare professionals to address ngā māuiui kai issues alongside gender-related goals in more responsive ways. Education about the risk and protective factors for ngā māuiui kai among trans people should be incorporated in relevant professional development and prevention initiatives to support responsive interventions and care in clinical and community settings.

Some limitations to our study are important to note. Due to the nonprobability community-based sampling, these findings are not representative of trans populations in Aotearoa. They may over-represent trans people who are connected to community networks or willing to share their experiences. While the SCOFF has been used and validated to measure eating disorder risk among trans people in previous studies (e.g., 19, 22), it does not account for symptoms associated with other aspects of ngā māuiui kai beyond restriction and binging. Like similar eating disorder screening tools, the SCOFF was developed to screen for risk among primarily young cisgender women. It may therefore be limited in its use among under-served populations who express ngā māuiui kai differently, including trans populations (49).

Effect sizes in the quantitative analyses were small and should be interpreted with caution. Due to the lack of open-text questions specifically about ngā māuiui kai, our analysis was limited to a small number of participants who shared their experiences of ngā māuiui kai in response to other questions about mental health. Data collection took place in late 2022, following the lifting of COVID-19 and some experiences with healthcare access or using eating as a way to cope with COVID-related stressors may reflect this broader context (e.g., 50). Increases in referrals to specialist eating disorder services in Aotearoa during COVID-19 (12, 51) suggest that ngā māuiui kai may have increased during this time, and these results could in part reflect this.

The New Zealand Eating Issues and Eating Disorders Strategy (51) outlines a vision where people and whānau (family) can “access treatment and supports that respond to their unique needs and contexts” (p. 1). Recommended actions in this strategy include to “[S]upport prevention initiatives that address the risk and protective factors associated with eating disorders” (p. 3), and to develop kaupapa Māori (Indigenous-led) services to improve access to care (p. 4). These recommendations combined provide specific opportunities to implement culturally and gender affirming practices for Māori trans people seeking care. Our findings support the rational for these initiatives by highlighting the increased risk of ngā māuiui kai among trans people who belong to other priority groups, and by showing the diverse contexts in which trans people experience ngā māuiui kai. Improving visibility of trans people’s experiences of ngā māuiui kai is critical for advancing equitable prevention and care.

## Data Availability

The dataset contains highly sensitive health information. We encourage researchers who wish to collaborate with the Counting Ourselves team to get in touch to request access to data. Requests to access the datasets should be directed to kiaora@countingourselves.nz.
