# Correction: Ngā māuiui kai: a cross-sectional study of elevated eating disorder risk and related experiences among trans people in Aotearoa

**DOI:** 10.3389/fpsyt.2026.1979757

**Published:** 2026-09-09

**Authors:** Alex Ker, Gloria Fraser, Micah Davison, Jaimie Veale

**Affiliations:** 1 School of Health, Te Herenga Waka – Victoria University of Wellington, Wellington, New Zealand; 2 Alumni of the School of Health Sciences, University of Canterbury, Christchurch, New Zealand; 3 School of Psychology, University of Waikato, Hamilton, New Zealand

**Keywords:** disordered eating, eating disorder, healthcare access, LGBTQIA, mental health, transgender

There was a mistake in **Table 1** as published. The table incorrectly stated the age categories for Youth as “14-18” and Adult as “19-54”. The corrected **Table 1** appears below.

**Table 1:** Sample characteristics of trans people in Aotearoa by SCOFF threshold, 2022 Counting Ourselves (n=2035).

Characteristic groups	*n* (%) within group who meets the SCOFF threshold (*n* = 697)	Effect size^a^	Chi-square^b^
Age		.124	***p* <**.**001**
Youth (14-24)	398 (39.4%)		
Adult (25-54)	282 (30.5%)		
Older adult (55+)	17 (16.8%)		
Gender		.044	*p=*.147
Trans man	161 (36.2%)		
Trans woman	142 (30.5%)		
Non-binary	385 (35.0%)		
Ethnicity^c^			
Māori	113 (44.1%)	.078	***p* <**.**001**
Pacific	19 (47.5%)	.039	*p=*.077
Asian	45 (31.3%)	-.018	*p=*.426
NZ European	518 (33.1%)	-.043	*p=*.055
MELAA or another ethnicity	14 (31.1%)	-.010	*p=*.654
Functional impairment (WGSS)	346 (46.9%)	.216	***p* <**.**001**
Deaf or disabled (self-identified)	224 (37.7%)	.043	p=.058
ADHD	310 (39.6%)	.087	***p* <**.**001**
Autism	286 (38.9%)	.066	***p=*.003**
Material hardship	459 (39.0%)	.138	***p* <**.**001**

^a^Cramer’s V was used to measure the effect size of gender and age (larger than 2x2 contingency tables). Phi was used to measure effect size of all other sociodemographic variables.

^b^Bold *p*-values indicate significant at *p ≤*0.05.

^c^Ethnicity was measured as a total count, rather than being prioritised. Each ethnicity category was tested compared to a binary reference group (ie. Māori, non-Māori).

The original version of this article has been updated.

